# A Rare Case of Methamphetamine-Induced Lung Injury During the Ongoing COVID-19 Pandemic

**DOI:** 10.7759/cureus.13215

**Published:** 2021-02-08

**Authors:** Anam Javed, Adeel Nasrullah, Khalid Malik

**Affiliations:** 1 Internal Medicine, Allegheny Health Network, Pittsburgh, USA; 2 Pulmonary and Critical Care Medicine, Allegheny Health Network, Pittsburgh, USA

**Keywords:** methamphetamine, ards, addiction medicine

## Abstract

Methamphetamine-induced lung injury is a very rare entity and is poorly understood due to the paucity of available literature. It can present with respiratory failure, often requiring immediate ventilatory support and conservative management. Secondary bacterial infection can result from smoking contaminated crystalline methamphetamine. Although there is growing evidence for the use of steroids in acute respiratory distress syndrome (ARDS), the literature is limited regarding cases of non-cardiogenic pulmonary edema due to meth use. We present a case of ARDS due to methamphetamine use, which dramatically resolved with ventilatory support. A low threshold to investigate drug-induced lung injury in suspicious cases can limit unnecessary utilization of resources during the ongoing coronavirus disease 2019 (COVID-19) pandemic.

## Introduction

Nearly 24 million people abuse crystal methamphetamine worldwide, and almost half a million Americans use methamphetamine every week [1]. It is associated with psychological and somatic health hazards such as inhalation lung injury and transmission of HIV and hepatitis B and C. Despite the increasing use of inhaled methamphetamine, its pulmonary complications are poorly understood due to the scarcity of available literature on the subject.

## Case presentation

**Figure 1:**
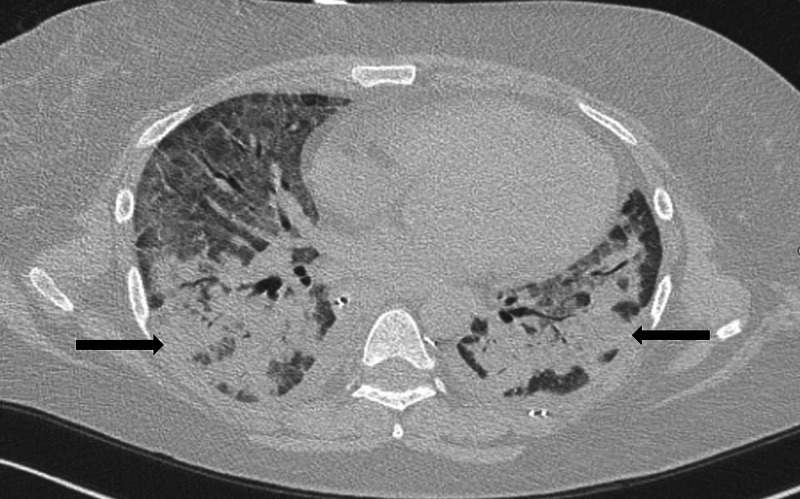
Bilateral ground-glass opacities seen on chest CT (black arrows) CT: computed tomography

A 45-year-old female with a past medical history of illicit drug use presented to the hospital with worsening shortness of breath. Two days prior, she had been treated with ceftriaxone and azithromycin for pneumonia at an outside facility. On presentation, she was afebrile, had a blood pressure of 128/80 mmHg, respiratory rate of 28 breaths/minute, and oxygen saturation of 87% on room air, requiring supplementary oxygen. Physical examination showed bilateral rhonchi, and decreased breath sounds were appreciated at the lung bases. She received methylprednisone and albuterol nebulizer therapy, but due to worsening hypoxia and work of breathing, she was intubated. Pertinent laboratory workup included a white blood cell (WBC) count of 26,000/ul with 91% neutrophils, hemoglobin of 8.8 g/dl, and procalcitonin of 0.34 ng/ml. A chest X-ray was obtained, which was concerning for acute respiratory distress syndrome (ARDS). CT scan of the chest was obtained for further characterization, which revealed diffuse ground-glass opacities with interlobular septal thickening (Figure 1).

She was treated empirically with vancomycin, cefepime, and azithromycin. Infectious workup returned negative for bacterial and viral etiologies while a urine drug screen came back positive for methamphetamines. On day two, a repeat test showed procalcitonin levels of 0.22 ng/ml, and hence antibiotics were discontinued. Due to a significant improvement in respiratory status, the patient was extubated on day two. On interval history, she admitted to smoking crystalline methamphetamine two days before admission. She was discharged home with the inclusion of health support for rehabilitation.

## Discussion

Acute use of oral or intravenous methamphetamine can cause psychosis, uncontrolled hypertension, acute coronary syndrome, hyperthermia, and disseminated intravascular coagulation (DIC) [2]. Crystalline methamphetamine is a widely smoked stimulant, which can cause a variety of pulmonary complications including ARDS, diffuse alveolar hemorrhage, eosinophilic pneumonia, and interstitial lung disease [3,4]. Due to its lipophilic nature, it is rapidly absorbed into the nasal mucosa and metabolized into active amphetamine, thus resulting in high blood concentration leading to more toxic effects as compared to oral or intravenous methamphetamine [2]. Although not studied in humans, animal studies have postulated free radical-related lung injury as a possible mechanism of pulmonary toxicity [5]. Chronic use of methamphetamine can lead to the remodeling of pulmonary arteries via smooth muscle proliferation resulting in pulmonary hypertension. It can present as nonproductive cough, dyspnea, and chest pain. The temporal relation of methamphetamine use, symptomology, exclusion of infectious causes, and radiographic findings such as ground-glass opacities can establish the diagnosis. Cessation of methamphetamine use and ventilatory and psychosocial support are core to management. Inhaled N-acetylcysteine, a free radical scavenger, might be useful while glucocorticoids have been proven to be efficacious in cases with eosinophilic pneumonia [5].

## Conclusions

In this report, we discussed a case of ARDS due to methamphetamine use that dramatically resolved with ventilatory support. We recommend considering methamphetamine inhalation injury in appropriate cases as early diagnosis and appropriate treatment can lead to a dramatic recovery.
